# Parent-to-parent support interventions for parents of babies cared for in a neonatal unit—protocol of a systematic review of qualitative and quantitative evidence

**DOI:** 10.1186/s13643-018-0850-2

**Published:** 2018-10-31

**Authors:** Harriet Hunt, Rebecca Whear, Kate Boddy, Leanna Wakely, Alison Bethel, Christopher Morris, Rebecca Abbott, Susan Prosser, Andrew Collinson, Jennifer Kurinczuk, Jo Thompson-Coon

**Affiliations:** 1 NIHR CLAHRC South West Peninsula (PenCLAHRC), University of Exeter Medical School, St Luke’s Campus, Exeter, EX1 1TE UK; 2 SNUG (Supporting Neonatal Users and Graduates) and Royal Devon & Exeter Hospital, Barrack Road, Exeter, EX2 5DW UK; 3 Royal Devon & Exeter Neonatal Unit, Royal Devon & Exeter Hospital, Barrack Road, Exeter, EX2 5DW UK; 4 Paediatrics and Neonatology, Royal Cornwall Hospitals NHS Trust, Treliske, Truro, Cornwall TR1 3LJ UK; 5 National Perinatal Epidemiology Unit, Nuffield Department of Population Health, University of Oxford, Oxford, OX3 7LF UK

**Keywords:** Neonatal, Parent support, Parent-to-parent, NNU, NICU, Preterm, Premature, Parent peer

## Abstract

**Background:**

Parents of babies admitted to neonatal units experience an arduous emotional journey. Feelings of helplessness, fear, sadness, guilt, grief and anger are common. These feelings can lead to anxiety, depression and post-traumatic stress which may persist long after discharge from the unit. Support from a parent with first-hand experience able to empathise with problems and challenges may help. This systematic review will identify quantitative and qualitative evidence to address the role of parent-to-parent support interventions for families of babies cared for in neonatal units, and combine the findings in an integrated synthesis.

**Methods:**

We are working in collaboration with a study-specific Parent Advisory Group (PAG) of parents who have relevant and varied lived experience of having a baby in neonatal care and those who have been involved in providing peer support. With the PAG, we will carry out a systematic review bringing together all existing research on parent-to-parent support for parents of babies cared for in neonatal units. This will be reported in accordance with the Preferred Reporting Items for Systematic Reviews and Meta-Analyses (PRISMA) statement. The protocol has been produced in accordance with the Preferred Reporting Items for Systematic Reviews and Meta-Analyses Protocol extension (PRISMA-P). We have co-produced a plain language protocol summary with the PAG which details the different stages of the project, and this is available via our website (http://clahrc-peninsula.nihr.ac.uk/research/parent-to-parent-support) for anyone interested in learning more about the detail of the project.

**Discussion:**

All outputs will be available on the NIHR CLAHRC South West Peninsula (PenCLAHRC) website and promoted via PenCLAHRC networks as well as organisations that have been contacted throughout the project. PAG members will be involved in writing and reviewing the academic paper and final report and in co-producing dissemination products such as plain language summaries. The PAG will influence the main conclusions of the systematic review, aid interpretation and help to communicate results in the most appropriate ways. We will hold an impact conference with representatives from neonatal units, national neonatal networks, commissioners of services and parents to discuss what the findings mean for clinical practice and service provision.

**Systematic review registration:**

PROSPERO CRD42018090569

**Electronic supplementary material:**

The online version of this article (10.1186/s13643-018-0850-2) contains supplementary material, which is available to authorized users.

## Background

### Description of the condition

Perinatal mental health problems carry a total economic and social long-term cost to society of about £8.1 billion for each 1-year cohort of births in the UK [1]. Improving support for parents of premature babies is an important priority for parents, carers, health care professionals and relevant charities. Two priorities identified at the 2014 James Lind Alliance Pre-Term Birth Priority Setting Partnership workshop [2] relate to improving support for parents: what should be included in packages of care to support parents and families/carers when a premature baby is discharged from hospital, and what emotional and practical support improves attachment and bonding and does the provision of such support improve outcomes for premature babies and their families.

The Picker Institute National Survey conducted in 2011 [3] also highlighted the need for improvements in parental support. Parents of babies admitted to neonatal units experience feelings of helplessness, guilt, failed expectations and uncertainty about their child’s prognosis. There is also uncertainty about the neonatal unit environment, what is and is not allowed and how best to interact with staff caring for their child. A parent may not feel welcome on the unit, may struggle to understand what is happening and may find it difficult to watch others care for their child [4].

Many parents experience practical difficulties associated with visiting their babies, looking after older siblings at home whilst their new-born is an inpatient and recurrent re-admissions. Fathers are often the sole source of support, and this can place additional stress on relationships. Unsurprisingly, there are higher levels of anxiety, depression and post-traumatic stress in mothers of pre-term babies than those whose children are born at term, and these mental health problems may persist long after discharge from the unit [5–8]. This, combined with high levels of parental stress, can affect the quality of the early parent-child relationship with long-term implications for the health of both the parent and child [4]. Reducing parent stress and improving self-esteem whilst on the unit and on taking their baby home can improve outcomes for parents (more positive parent-child interaction and improved parent confidence) and their children (improved cognitive outcomes [9], language development [10]), reduce the length of stay in neonatal units and reduce re-admissions [11–13].

### Description of the intervention

The Toolkit for High Quality Neonatal Services [14] states that well-organised, effective and sensitive neonatal care can make a lifelong difference to premature and sick newborn babies and their families. Getting this early care right is the responsibility of the NHS. One of the principles of care described is the need for a family-centred philosophy of care that helps families whose baby is in hospital to cope with the stress, anxiety and altered parenting roles that accompany their baby’s condition [14], and these sentiments are reflected in the new service specification for neonatal care. A variety of different interventions have been developed to support parents at this difficult time and to encourage and involve them in the care of their baby [15]. One of these interventions is peer support, defined as the provision of emotional, appraisal and informational assistance by a selected social network member who possesses experiential knowledge of a specific behaviour or stress and similar characteristics as the target population [16]. Being able to talk to someone (a parent) who is familiar with the experience of life in the neonatal unit can be uniquely beneficial. Some studies have suggested that key benefits include the greater perceived empathy that peer supporters are seen to have for the individuals they support, and opportunities for parent empowerment and improved relationships with healthcare workers within an environment that is rarely parent centred [15, 17]. The intervention of interest within this systematic review is specifically parent-to-parent (P2P) peer support, defined by the study authors as peer support provided to parents by parents (with further support provided by a wider network if applicable).

The support received by families within the neonatal unit can be as varied and individual as the families themselves [18]. A number of different support models exist including individual support, parent support groups and internet-based groups [18]. This review will document types of parent-to-parent support interventions within included studies using the Template for Intervention Description and Replication (TIDieR) checklist [19]. Through this systematic review, we aim to find out whether parent-to-parent support is helpful for families experiencing neonatal care; what this support looks like; and what factors can help or prevent parent-to-parent support being available.

### How the intervention might work

Being able to talk to someone (a parent) who is familiar with the experience of pre-term birth, life in the neonatal unit, a child born with special health care needs, loss of pregnancy and/or infant loss may help to reduce feelings of isolation, anxiety, stress and depression. Peer support has been shown to help people, in aspects such as depression, stress, post-traumatic stress disorder, emotional support and isolation, with a variety of health conditions [20–23]. The UK Department of Health and Social Care is promoting increased roles for the voluntary, community and social enterprise sector in delivering health and social care [24] and peer support may be a cost-effective option [25]. In our recent systematic review of peer support for parents of disabled children, qualitative studies consistently suggest that recipients of peer support perceive a benefit, an effect seen across different types of medical conditions [26–29]. Research also suggests benefits for being a peer supporter including increased confidence, self-esteem and facilitating their own recovery [22]. There have been previous reviews of support interventions for families in neonatal units although peer support interventions have only been a small part of their scope and, crucially, have not covered parent-to-parent support as an intervention that might continue after discharge from the neonatal unit [15]. Recent scoping searches suggest that since these reviews there has been an increasing research interest and momentum for the role of P2P support in neonatal units [18] which needs consolidating.

### Why it is important to do this review

This systematic review will be the first, to our knowledge, to identify both quantitative and qualitative information to address the role of parent-to-parent support interventions for families of babies cared for in neonatal units, and combine the findings in an integrated synthesis. The integration of quantitative and qualitative information from published and unpublished sources will enhance the utility and impact of the review, allowing greater exploration and understanding of the results. Stakeholders from a variety of perspectives (e.g. medical, nursing, parent, peer-support volunteers and the NHS neonatal network) will be involved in co-producing the review ensuring that the results are relevant and applicable to the context of current provision and our target audiences [30]. We have engaged with key stakeholders and end-users of this research to develop a communication and dissemination plan that will ensure the findings reach those who can use them to improve care and outcomes for families experiencing and transitioning out of neonatal care [the Communication and dissemination plan is available in Additional file 1].

## Methods

### Patient and public involvement

The systematic review will be conducted in collaboration with a study-specific Parent Advisory Group (PAG). Group members will include parents who have relevant and varied lived experience of having a baby in neonatal care and those who have been involved in providing peer support. Four face to face meetings will take place over the course of the study. In addition, parents will have opportunities to be involved in stages of the project as their circumstances allow.

The PAG will contribute to the protocol development by involvement in finalising the research questions, ensuring outcomes are meaningful to parents as well as professionals, refining the inclusion/exclusion criteria and helping with search terms. There will be opportunities for the group to undertake tasks (with full support and training) within the review process to the extent that they are willing or able to commit their time, e.g. screening papers for inclusion, reading and commenting on papers selected for inclusion, interpreting findings and contributing to the qualitative synthesis.

The PAG will be involved in writing and reviewing the academic papers and in co-producing dissemination products such as plain language summaries.

### Objectives

#### Main objective

To bring together studies which have explored the experience of P2P support from the perspective of the persons giving and receiving P2P support, or those involved in implementing P2P support in the context of the provision of neonatal care.

#### Secondary objectives

1. To determine the key characteristics, resources, components and processes that should be included in P2P support for parents of babies cared for in neonatal care settings, whilst on the unit and after discharge home.
2. Where adequate data are available, to determine the effectiveness and cost-effectiveness of P2P support interventions on the health and well-being of parents and their babies.
3. To explore what the findings mean for clinical practice and service provision.

### Eligibility criteria

We will carry out a systematic review, which brings together all existing research on parent-to-parent support. This will be reported in accordance with the Preferred Reporting Items for Systematic Reviews and Meta-Analyses (PRISMA) statement [31]. The protocol has been produced in accordance with the PRISMA-P reporting guidelines for systematic review protocols [32] [see completed PRISMA-P checklist in Additional file 2]. The protocol is registered on the International Prospective Register of Systematic Reviews (PROSPERO), registration number CRD42018090569. A plain language protocol summary has been co-produced with the PAG and is available in Additional file 3.

Studies will be included if they describe the effects of P2P support and report data on one or more outcome measures. We will not restrict by study design but will stratify the reporting of results prioritising the most robust evidence. Qualitative studies that do not include a comparison group will not be excluded but will be used to inform the experiences reported by those offering and receiving P2P support where applicable.

#### Study reports

Editorials, opinions and letters will be excluded. Authors of studies published only as abstracts will be contacted and asked to provide further detail. If no further detail is available, the study will be excluded. Studies may compare support with no support or compare one type of support with another. The inclusion/exclusion criteria will be tested through piloting by two reviewers to establish agreement prior to commencing the study selection process. We will consider the relevance of study setting to the UK health care system. However, we believe that elements of P2P support provision in other health care settings may be transferable to the UK health care system. Only qualitative papers written in English will be included to avoid loss or distortion by translation from studies written in another language. Non-English language quantitative papers will be translated.

### Participants

The population of interest is parents of babies cared for in neonatal units accessing the support service at any time (during their time in hospital or back home in the community). We will focus on P2P support interventions for those with experience of neonatal care (where those providing the support may be volunteers or paid parent workers).

### Interventions

Studies will be included if they describe the effects of P2P support and report data on one or more outcome measures. We will exclude studies relating to interventions offered by professionals or interventions which offer instruction or training to parents rather than support, studies of peer support specifically for families affected by bereavement or for those whose babies are receiving palliative care and studies which do not adequately describe the intervention. It may not be possible to describe the ‘content’ of the intervention as conversations are individualised and private and do not necessarily follow a formal predefined structure. Adequate description of the ‘context’ of the intervention will therefore be sufficient including description of both parties, level of training of the person offering support, extent of support being offered in terms of time and availability and a description of the purpose of the intervention, for example emotional support, information and practical support. Efforts will be made to contact the authors of relevant studies for further clarification if the description is not sufficient before the study is excluded.

Studies may compare support with no support or compare one type of support with another.

### Outcomes

#### Role of outcomes

Studies that report (quantitatively or qualitatively) at least one of the following outcomes will be included:

- Experiences of people offering and receiving P2P support, including, but not solely, self-confidence, self-efficacy, knowledge, moving onto or back into employment, sense of belonging/friendship/being part of something
- Measures of parent-child interactions; parent health, e.g. stress, depression, confidence, isolation, satisfaction and empowerment; child health and development, e.g. cognitive development, behaviour and language; family function including relationships and transitioning home; service use, e.g. readmissions and contact with other services; and resource use where reported
- Contextual factors and modifiers to implementation of P2P interventions
- Adverse outcomes
- Effects on practice

#### Outcome domains of interest

Primary outcomes will include the experiences of people offering, receiving and implementing P2P support, for example outcomes relating to self-confidence, guilt, self-efficacy, knowledge, moving onto or back into employment, sense of belonging/friendship/being part of something, reassurance of shared experiences, and socialising with others who have similar experiences, agency and self-help, feeling of the baby belonging to the parent (or not), navigating the system, and managing differences between units.

Secondary outcomes will include measures of:

- Parent-child interactions, ownership (e.g. “feeling like it’s your baby”)
- Parent health e.g. stress, depression, guilt, confidence, isolation, satisfaction, empowerment;
- Child health and development e.g. cognitive development, behaviour, language;
- Family function including partner relationships, sibling relationships, mental health, transitioning between units and moving home;
- Service use e.g. readmissions, contact with other services;
- Resource use;
- Potentially negative impacts of P2P support such as increased anxiety, learning details from others that were not expected/wanted or frightening;
- Bad experiences of being a peer supporter such as the impact of a negative event (e.g. the death of a child).

#### Outcome measures of interest

It is likely that different measures and time points will be reported across studies. It is important that the variability is captured in the context of this review; therefore, specific measures/time points are not highlighted here. Where possible, these will be analysed together but where the measures and/or time points are greatly varied these results will be described and discussed narratively. 

### Minimally important difference

Due to the nature of this review, it is not thought that pre-specified minimally important differences are appropriate, though the relevance and potential impact of any differences in effects will be discussed.

### Search methods

#### Search sources

The search strategy will cover both electronic bibliographic database searching and supplementary search methods to capture both published and unpublished (grey) literature. The database search will be designed by an information specialist (AB) using a combination of controlled vocabulary (e.g. MeSH) and free text terms. The PAG will be consulted to ensure unusual terminology is included. No language, date or study type limits will be applied. The bibliographic databases to be searched will include MEDLINE, EMBASE, PsycINFO, CINAHL, Social Policy and Practice (SPP), The Cochrane Library (CDSR, CENTRAL and DARE), Applied Social Sciences Index and Abstracts (ASSIA), British Nursing Index (BNI), Social Science Citation Index (SSCI), Health Management Information Consortium (HMIC), PQDT, Explore and Midwives Information and Resource Service.

#### Supplementary search

Supplementary search methods will include citation chasing using SCOPUS and Web of Science, hand searching of relevant journals, web searching, searching of University repositories and searching organisations websites: The National Childbirth Trust, BLISS, The Royal College of Midwives, Neonatal Nurses Association, the National Association of Neonatal Nurses, Bonnie Babies, Tommy’s, the Royal College of Obstetricians and Gynaecologists, the Royal College of Paediatrics and Child Health, the British Association of Perinatal Medicine, the Neonatal Nurses Association and the Neonatal Society. We will also search for reports from the voluntary sector, and local initiatives from primary care trusts (PCTs) that offer P2P support, and university repositories that specialise in Masters of Nursing courses, and will consult stakeholders for suggestions of additional websites.

#### Search strategy

The main search will include population terms: (neonatal OR premature OR preterm) and (baby OR babies OR infant) and intervention terms: peer support OR support network OR (mother OR father OR parent OR maternal OR paternal) AND (support OR network). Database syntax (e.g. adjacency searching) and additional synonyms will be used where relevant [an example search strategy is shown in Additional file 4].

These terms will be discussed with the project team and PAG before confirming the full search strategy to ensure all relevant terms are included to the best sensitivity and specificity of the search to ensure appropriate use of research resources. This main strategy will then be adapted for other databases.

We will use iterative searches to explore where necessary. For instance if named interventions are identified as being particularly relevant, further citation chasing, web-based or database searches may be carried out and the audit trail will reflect this. Search results will be interrogated to ensure that key papers are identified.

#### Search restrictions

No restrictions will be used in the search for literature.

### Data collection and analysis

#### Inclusion decisions

The abstracts and titles of references retrieved by the electronic searches will be screened independently by two reviewers (out of HH, RA, KB and AB) using the pre-specified inclusion/exclusion criteria. The full text of potentially relevant studies will be obtained. Using the same methods, full text articles will be assessed for inclusion by two reviewers (HH, RA, KB and AB). Any quantitative papers in non-English languages will be translated.

Discrepancies will be resolved by discussion, with a third reviewer (JTC) if necessary. We will exclude duplicate papers. A PRISMA-style flowchart will be produced detailing the study selection process with the reason for exclusion of each full-text paper reported.

#### Data collection process

Quantitative data will be extracted by one reviewer (HH) into a piloted, standardised data extraction form. This will be checked by another reviewer (RA). Discrepancies will be resolved by discussion with the involvement of a third reviewer (JTC) if necessary. We will use the template for intervention description and replication (TIDieR) checklist [19] to inform the systematic collection of this information.

For qualitative studies, we will extract details of the study aim, the sample, and the type and nature of the intervention/programme. We will also collect data on the theoretical approach, the methods used to collect the data, the analytic processes and the quotes, themes and concepts pertinent to our research questions. This process will be conducted by two reviewers independently (HH plus RA). Any discrepancies will be resolved through discussion. A structured summary will be produced for each paper and the extracted data will be tabulated allowing comparison between studies.

#### Requests for data

Where papers provide insufficient details of the intervention, e.g. what is delivered and by whom, we will contact authors to obtain unpublished details.

#### Data items

The following data will be tabulated: details of the intervention type and content; study date and publication date; the setting and the provider; sample characteristics of the included population; and the type of outcomes measured.

#### Missing data

Missing data will be identified and recorded within the review. Where means and standard deviations are not available, we will collect effect estimates, confidence intervals, test statistics, *P* values and individual participant data where reported. Authors will also be contacted for clarification of any missing data.

### Tools to assess quality

We will use appropriate quality assessment tools depending on the design of the included studies using the guidance produced by the NHS Centre for Reviews and Dissemination [33] and the Cochrane Collaboration.

### Risk of bias assessment process

The risk of bias of individual studies will be assessed using a suitable tool by one reviewer (HH), checked by a second reviewer (RA), and any disagreement will be resolved by discussion involving a third reviewer (JTC) if necessary. We will include all reported effect measures used to describe effect sizes in included studies and meta-analyses (e.g. risk ratio or odds ratio, mean difference or standardised mean difference). We will make a judgement on the most appropriate measures for synthesis depending on availability and appropriateness of available data, and clearly document our decision-making processes.

### Quantitative synthesis

We will use methods of systematic review and evidence synthesis as outlined by the Cochrane Handbook [34]. Quantitative data will be tabulated and discussed narratively in the first instance. If sufficient quantitative data are located, appropriate meta-analysis will be used to synthesise the effectiveness of similar interventions using a random effects model. It is not expected that enough RCTs will be found to conduct specific analyses of heterogeneity or other sub-group analyses (but potential sub-group analyses could involve, e.g. parent-to-parent peer support versus other forms of peer support).

#### Unit of analysis issues

As we will not restrict by study design, we will take a pragmatic approach to addressing clustering, matching, double counting or other design features of the included studies using the guidance produced by the NHS Centre for Reviews and Dissemination [33] and the Cochrane Collaboration.

#### Studies with more than two groups

It is considered unlikely that studies will be identified with multiple arms. If studies with more than one study arm are identified and included within our review, we will analyse multiple intervention groups in an appropriate way that avoids arbitrary omission of relevant groups and double-counting of participants using the guidance produced by the NHS Centre for Reviews and Dissemination [33] and the Cochrane Collaboration.

### Qualitative synthesis

We will extract details of the methods of each study along with quotes, themes and concepts pertinent to our research questions. Structured summaries will be produced for each paper. This is produced so that the same information, recorded in the same order, is given for each study. To facilitate this step, a number of additional strategies may be used, such as grouping the studies according to elements such as study design, the type of intervention under study, its context, populations and types of outcome measures and so on. Tabulation can also assist synthesis and may be needed to produce the initial descriptions. The findings of each qualitative paper will be coded and grouped together into broad themes through discussion. The PAG will be invited to a qualitative synthesis reference group meeting to discuss the qualitative synthesis to establish relevance and transferability to a wider audience. This meeting may be followed up with emails and telephone conferences to explore themes and ideas further.

### Narrative synthesis

We will use formal methods of narrative synthesis outlined by Rodgers and colleagues to bring together the findings of the qualitative and quantitative reviews [35]. Narrative synthesis is a method of synthesising quantitative and qualitative findings about effectiveness *and* implementation and relies on text to “tell the story” of such findings. A number of possible tools and techniques are offered as mechanisms for manipulating, analysing and comparing the results of studies included in a review. We will use the descriptive paragraphs produced during the synthesis of the qualitative information and numerical information presented in a common rubric (where possible). Various techniques can be used to explore within-study and between-study relationships. Visual tools include ideas webbing (spider diagrams) and concept mapping (using diagrams and flow charts to represent relationships between and within studies that are being explored by the reviewer), and textual case descriptions, and triangulation (to consider how methodological and theoretical approaches may have impacted on the outcomes). The exact choices of methods will be developed through the synthesis process and reasons for our choices recorded and reported.

### Risk of reporting bias across studies

In line with current advice on the dangers of misinterpretation [33] in the event that we identify statistical heterogeneity and small studies, we will not produce funnel plots to explore potential reporting bias across studies. This would be due to their inappropriateness where statistical heterogeneity is present, where studies are under-powered or there are small numbers of studies being assessed.

### Addressing risk of bias

It is likely that we will encounter bias due to the nature of the studies within the inclusion criteria. We will assess the impact of risk of bias using appropriate methods according to the guidance produced by the NHS Centre for Reviews and Dissemination [33] and the Cochrane Collaboration.

### Subgroup analyses

It is unlikely that data will be available for sub group analyses, and we have not pre-planned any subgroup analyses.

### Methods for qualitative research evidence

Findings from each qualitative study will be synthesised using the methods described above. We hope to develop an explanatory synthesis to explain the effectiveness of P2P support interventions for the health and well-being of parents and their premature babies, and explain what these findings mean for clinical practice and service provision.

### Quality of the evidence

The current GRADE guidance will be used to assess the quality of quantitative evidence [36]. GRADE-CERQual will be used to assess confidence in the findings of the qualitative evidence synthesis (http://journals.plos.org/plosmedicine/article?id=10.1371/journal.pmed.1001895).

### Summary of findings table

After review by our PAG, the outcomes to be reported in the summary of findings table will be those relating to the primary outcomes of personal experiences of people offering and receiving P2P support (including, but not solely, self-confidence, self-efficacy, knowledge, moving onto or back into employment, sense of belonging/friendship/being part of something); measures of parent-child interactions; parent health, e.g. stress, depression, confidence, isolation, satisfaction, empowerment; child health and development (e.g. cognitive development, behaviour, language); family function including relationships and transitioning home; service use (e.g. readmissions, contact with other services); and resource use where reported.

The most appropriate effect measures reported for each outcome will be selected depending upon included study designs.

## Discussion

### Dissemination and impact

The project team has worked with the PAG to develop a communication and impact strategy to effectively communicate review findings, which includes presenting research findings at academic and clinical conferences and within open access peer reviewed journals [available in Additional file 1]. We have produced a plain language summary of the project to help inform and recruit the PAG [available in Additional file 5]. A plain language protocol summary has been co-produced with the PAG to detail the different stages of the project, what steps will be completed when and how they fit within the context of the whole project [this is available in Additional file 3].

All outputs will be available on the PenCLAHRC website and promoted via PenCLAHRC networks as well as organisations that have been contacted throughout the project.

There will be three main routes to ensuring research findings are meaningful in practice and that they reach the people they are most concerned, i.e. parents of babies receiving neonatal care and the people who care for them.

These routes are:

#### The Parent Advisory Group

As well as contributing to the development of this protocol, PAG members will have the opportunity to carry out tasks within the systematic review (such as screening papers for inclusion and interpreting findings). Via PAG meetings and a closed Facebook group, PAG members will be involved in writing and reviewing the academic paper and final report to NIHR and will be involved in co-producing dissemination products such as plain language summaries. The PAG will therefore help to disseminate findings by influencing the main conclusions of the systematic review, aiding interpretation and helping to communicate results in the most appropriate ways.

#### Phone calls with South West Neonatal units

Two review team members (SP assisted by HH) will conduct calls to the relevant contacts in each of the 12 South West Neonatal units to establish what (if any) parent support services are provided on their unit, what those services look like and who provides them, what barriers or facilitators have been seen in implementation and any concerns about service sustainability.

The PAG reviewed the draft questions and contributed a number of additional questions and prompts to be included in the topic guide and question list for these phone calls.

The aim is to identify key messages for dissemination, to enhance the ability of the research findings to be to be useful in the context of the current parent support services and to provide positive and lasting engagement between the research findings and neonatal units [the topic guide for these phone calls is provided in Additional file 6].

#### Impact conference

We will convene an Impact Conference, which will be a 1-day event with representatives from neonatal units, national neonatal networks, commissioners of services and parents with experience of neonatal unit life to discuss what the findings mean for clinical practice and service provision. The aims of the impact conference will be to disseminate our findings directly to those who are able to use them to make a difference to the lives of parents and their babies; explore our findings in the context of existing provision of P2P support from the perspectives of those receiving, delivering and providing services; explore potential barriers to the successful implementation of effective P2P support interventions and suggest co-developed solutions; ensure our findings are interpreted and reported using language useful for practice within the NHS; and to highlight priorities for further research in this area.

The PAG will play a pivotal role in the Impact Conference and subsequent dissemination of research findings, having the knowledge of and contacts within the relevant parent and professional organisations.

### Changes to the protocol

Any deviations from the protocol will be documented in the final report and published manuscripts.

## Additional files

Additional file 1: Communication and dissemination plan. (DOCX 25 kb)

Additional file 2: PRISMA-P checklist. (DOCX 29 kb)

Additional file 3: Plain Language Protocol Summary. (DOCX 6162 kb)

Additional file 4: Example Search Strategy. (DOCX 16 kb)

Additional file 5: Plain language summary. (PDF 315 kb)

Additional file 6: Fact finding phonecalls topic guide. (DOCX 21 kb)

## Abbreviations

ASSIA: Applied Social Sciences Index and Abstracts
BLISS: UK charity for babies born premature or sick
BNI: British Nursing Index
CDSR: The Cochrane Database of Systematic Reviewss
CENTRAL: Cochrane Central Register of Controlled Trials
CINAHL: Cumulative Index of Nursing and Allied Health Literature
DARE: Database of Abstracts of Reviews of Effects
EMBASE: A biomedical and pharmacological database of published literature
GRADE: Grading of Recommendations Assessment, Development and Evaluation
GRADE-CERQual: Confidence in the Evidence from Reviews of Qualitative Research
HMIC: Health Management Information Consortium
MEDLINE: A bibliographic database of life sciences and biomedical information
MeSH: Medical Subject Headings
NHS: National Health Service (UK)
NICU: Neonatal Intensive Care Unit
NIHR: National Institute for Health Research
NNU: Neonatal Unit
ORCiD: A nonproprietary alphanumeric code to uniquely identify scientific and other academic authors and contributors
P2P: Parent-to-parent
PAG: Parent Advisory Group
PCT: Primary Care Trust
PenCLAHRC: NIHR CLAHRC South West Peninsula
PQDT: ProQuest Dissertation & Theses
PRISMA: Preferred Reporting Items for Systematic Reviews and Meta-Analyses
PRISMA-P: Preferred Reporting Items for Systematic Reviews and Meta-Analyses – Protocol extension
PROSPERO: International prospective register of systematic reviews
PsycINFO: Abstracting and indexing database covering behavioral sciences and mental health
RfPB: Research for Patient Benefit
SCOPUS: Elsevier’s abstract and citation database
SNUG: Supporting Neonatal Users and Graduates
SPP: Social Policy and Practice
SSCI: Social Science Citation Index
TIDieR: Template for Intervention Description and Replication
