# Supplementary material for: Parent-to-parent support interventions for parents of babies cared for in a neonatal unit—protocol of a systematic review of qualitative and quantitative evidence
Source: Syst Rev. 2018 Oct 31;7:179. doi: 10.1186/s13643-018-0850-2 (PMC6211448; doi:10.1186/s13643-018-0850-2)
Supplement: Supplementary file 1 — Communication and dissemination plan. (DOCX 25 kb) [file 13643_2018_850_MOESM1_ESM.docx]

## Communication and dissemination plan

PaReNt project

Communication and dissemination plan

Activities

1. [Parent Advisory Group](#one)
2. [Fact-finding phone calls](#two)
3. [Parent-guided activities](#three)
4. [Impact Conference](#four)
5. [Networks](#five)
6. [Conference presentations](#six)
   1. British Association of Perinatal Medicine (BAPM) conference - 27/28 September, Leeds (UK)
   2. Cochrane Colloquium – 16-18^th^ September, Edinburgh (UK)
7. [Full project report for NIHR](#seven)
8. [Peer reviewed open access journal publication](#eight)
9. [Project protocol](#nine)
10. [Plain language protocol summary](#ten)
11. [Plain language research summaries](#eleven)

1. [Podcast](#twelve)
2. [Value for money outputs](#thirteen)
3. [Timeline & Gannt chart](#fourteen)
4. Parent Advisory Group (LW/KB/CM leading)

The Parent Advisory Group (PAG) plays a pivotal role in the research project. Specifically, the PAG will help to shape the Impact conference and subsequent dissemination of research findings, having the knowledge of and contacts within the relevant parent and professional organisations. PAG input and consultation will be via meetings and a closed Facebook group with low impact administration by LW.

The PAG will be invited to a qualitative synthesis reference group meeting to discuss the qualitative synthesis to establish relevance and transferability to a wider audience. This meeting may be followed up with emails and telephone conferences to explore themes and ideas further.

The PAG held its first meeting on Tuesday January 30^th^ attended by LW, KB, HH, HH and parent members. There will be another 2 meetings throughout the project in June and September (tbc).

1. Short ‘fact-finding’ phone calls (conducted/supervised by SP)

SP will make the phone calls using her professional network of staff from neonatal units within the South West.

HH will assist by note taking and clarifying any project details during or after the phone calls. We have allocated two timeslots to make these calls on Thursday 22^nd^ February 1-4pm and Monday 26^th^ February from 11.00 to 1.30pm in Sue’s office at the RD&E in Exeter.

What’s the aim of these phone calls?

We will talk to the relevant contacts in each of the 12 South West Neonatal units to establish

- what (if any) parent support services are provided on their unit,
- what those services look like,
- who provides them,
- what barriers or facilitators have been seen in implementation and
- any concerns about service sustainability.

How will this information be used?

We will use this work alongside any other similar work conducted in the UK to more fully inform the discussion during the impact conference and the subsequent key messages to be promoted from the research findings.

We will be in touch with our contacts from each of the 12 units to let them know how the project is progressing, and to invite people along to our South West impact conference in July/September 2018. The aim of this is to discuss what we have found, what the really important messages are and how to get these out to where they are most needed.

1. Parent-guided activities

Central to the ethos of this project is the meaningful involvement of parents, brought together within the project through project team membership (Leanna Wakeley representing SNUG) and the Parent Advisory Group (PAG). We will be guided by the PAG about appropriate dissemination avenues and as part of this process will take an inductive approach to communication and dissemination of findings. In practice this means that we are not sure what this will look like, but may use e.g. social media, targeted blogs/websites/forums that the PAG recommends as places where we need to publicise our findings. We will work with the PAG to create a list of the most useful places and work with them to target the message we want to put out. We will do this partly by using the Facebook group and partly face to face during our scheduled PAG meetings.

1. Impact conference

We will hold a one day conference in the South West (Exeter area) July/September 2018 to discuss our emerging results, talk about ways forward for implementation, and explore evaluation in the context of the current service provision as determined by the fact-finding phone calls or other existing information on service provision locally and/or nationally based if this has already been conducted.

We will invite representatives from:

- each of the 12 neonatal units in the South West;
- relevant charities, hospitals and professional organisations such as BLISS (from whom we have a letter of support);
- the South West Neonatal Network (RL);
- the Neonatology subsection of the Royal College of Paediatrics and Child Health
- the National Perinatal Epidemiology Unit (and others),

alongside all members of the PAG.

Impact Conference aims

The aims of the impact conference will be to:

- disseminate our findings directly to those who are able to use them to make a difference to the lives of parents and their babies,
- explore our findings in the context of existing provision of P2P support from the perspectives of those receiving, delivering and providing services,
- explore potential barriers to the successful implementation of effective P2P support interventions and suggest co-developed solutions,
- ensure our findings are interpreted and reported using language useful for practice within the NHS and
- to highlight priorities for further research in this area.

1. Networks

Representatives from our target audiences will work with us to ensure that we interpret and present the findings in a series of unbiased dissemination products which can be used effectively by the four key groups of decision makers.

- 1. Policy makers (national and local). Contacts within Public Health at Devon County Council and the facilitator of the local Maternity Services Liaison Committee have agreed to help identify opportunities for dissemination to local NHS commissioners, ensuring that study findings are immediately available to the local NHS to inform decision making. The National charity BLISS have agreed support to disseminate the findings via their channels accessed by policy makers.
  2. Service providers (clinical and non-clinical). In order to engage with clinical service providers nationally, findings will be presented at the Royal College of Paediatrics and Child Health (RCPCH) annual conference. We will also publish the findings of the systematic review in a high ranking peer reviewed journal relevant to the intended audience and consider summary articles for journals such as Infant or Journal of Neonatal Nursing as recommended. Co-applicants SP and AC alongside Rebecca Lemin (from South West Neonatal Network) will help to identify opportunities to present and discuss the findings with those involved with service provision.
  3. Members of the public. Plain language summaries and a podcast will be coproduced with charities and families. Parents and families will also help to identify appropriate social media platforms for sharing the findings.
  4. Researchers. Methodological and topical knowledge will be shared at the RCPCH conference and Cochrane colloquium and in promoting the peer reviewed journal publication within relevant networks.

1. Conference presentations
   1. Royal College of Paediatrics and Child Health (RCPCH) Conference - 27/28^th^ September, Leeds (UK)

In order to engage with clinical service providers nationally, we will present the findings at the British Association of Perinatal Medicine (BAPM) conference taking place on 27/28 September, Leeds (UK). This is conference focussed towards clinicians and healthcare professionals, and we have budgeted for travel & subsistence, and accommodation for 2 nights for 2 people.

- 1. Cochrane Colloquium – 16-18^th^ September, Edinburgh (UK)

This year there is no NIHR INVOLVE conference, which would have been our first choice for involving parents in presenting the research. Instead we have decided to target the Cochrane Colloquium being held in Edinburgh in September, as the explicit focus of the conference is to run an event that is “co-designed, co-produced and co-presented by healthcare consumers and where everyone’s input is valued equally” (<http://community.cochrane.org/news/cochrane-colloquium-edinburgh-2018>). We would like to give the parents and families an opportunity to disseminate the findings from their perspective. We have therefore budgeted for travel & subsistence, and accommodation for 1 night for 3 people.

The call for Abstracts closes on 2^nd^ March, and we have 4 abstracts planned for submission:

1. Search terms for PaReNt – AB/KB
2. PPI meetings in non-meeting rooms – PaReNt plus other experience – KB
3. Reflections on embedding PPI in PaReNt – HH
4. Plain language protocol summary – PaReNt – JTC
5. Full project report for NIHR

We will produce a full project report for NIHR as agreed in our funding bid.

1. Peer reviewed open access journal publication

Open access fees of £2500 allocated, and we have secured additional agreement from the University of Exeter to fund open access for our protocol. We will aim to publish our protocol in the Systematic Reviews journal.

1. Project protocol

We will produce a technical protocol for open access publication in a peer reviewed journal (BMC Systematic Reviews is the target journal). This will detail the research authors, background to the project, Parent Advisory Group, objectives, eligibility criteria, search methods, data collection and analysis, dissemination and impact, acknowledgements, contributions of authors, conflict of interest statements, sources of support and appendices. Within the appendices will be a copy of the plain language summary protocol (see below) and detailed search strategy.

1. Plain language protocol summary (HH/LW/CM/KB)

This is a plain language version of the protocol in summary form, and is intended to allow anyone to understand how we plan to conduct our systematic review and other research activities regardless of specialist knowledge. This will be co-produced with the PAG via our project team parent and charity representative LW and via our closed Facebook group. The document will be available within the appendices of the project protocol, published on our project website and available alongside the final report to the NIHR. To our knowledge, this is the first plain language protocol document produced and as such, we will reflect on the usefulness of this document with the PAG via our quarterly meetings. We will submit an abstract to present our reflections at the Cochrane Colloquium in September 2018.

1. Plain language research summaries

Plain Language research summaries will be produced by the end of September 2018. These summaries will be guided by the PAG and will summarise results targeted to a particular stakeholder group. Printing costs of £500 have been allocated.

1. Podcast

The podcast will be developed with the project team and PAG input, for dissemination alongside the Impact Conference and other dissemination activities.

1. Value for money outputs

A full report of the project will be submitted to the NIHR, full findings will be published in an open access peer reviewed academic journal prepared according to PRISMA guidelines and presented at conferences. Audience-specific, targeted findings summaries will be co-produced and co-disseminated with the help of individuals from each target audience. An impact conference will be held to engage locally and nationally relevant clinicians and service providers and parents with the findings, with the aim of using even broader experiences to interpret the findings and helping the messages of the research to be used to inform practice and provision of parent-to-parent support services in the neonatal environment.

1. Timeline and Gannt Chart

| 2018 | Jan | Feb | Mar | Apr | May | June | July | Aug | Sept | Oct | Nov | Dec |
| --- | --- | --- | --- | --- | --- | --- | --- | --- | --- | --- | --- | --- |
| Month | 1 | 2 | 3 | 4 | 5 | 6 | 7 | 8 | 9 | 10 | 11 | 12 |
| Parent Advisory group meeting/workshop |  |  |  |  |  |  |  |  |  |  |  |  |
| Project management meeting |  |  |  |  |  |  |  |  |  |  |  |  |
| Qualitative findings meeting (PAG) |  |  |  |  |  |  |  |  |  |  |  |  |
| Finalise protocol and plain language protocol summary |  |  |  |  |  |  |  |  |  |  |  |  |
| Design & run search strategy |  |  |  |  |  |  |  |  |  |  |  |  |
| Title and abstract screening |  |  |  |  |  |  |  |  |  |  |  |  |
| Short phone surveys |  |  |  |  |  |  |  |  |  |  |  |  |
| Full text retrieval |  |  |  |  |  |  |  |  |  |  |  |  |
| Additional searching |  |  |  |  |  |  |  |  |  |  |  |  |
| Full text screening |  |  |  |  |  |  |  |  |  |  |  |  |
| Citation chasing |  |  |  |  |  |  |  |  |  |  |  |  |
| Data extraction |  |  |  |  |  |  |  |  |  |  |  |  |
| Quality appraisal |  |  |  |  |  |  |  |  |  |  |  |  |
| Data checking |  |  |  |  |  |  |  |  |  |  |  |  |
| Synthesis |  |  |  |  |  |  |  |  |  |  |  |  |
| Prepare draft paper |  |  |  |  |  |  |  |  |  |  |  |  |
| Prepare paper for submission to peer reviewed journal |  |  |  |  |  |  |  |  |  |  |  |  |
| Co-produce plain language summaries |  |  |  |  |  |  |  |  |  |  |  |  |
| Edits and dissemination |  |  |  |  |  |  |  |  |  |  |  |  |
| Prepare communication and impact strategy |  |  |  |  |  |  |  |  |  |  |  |  |
| Impact conference |  |  |  |  |  |  |  |  |  |  |  |  |
| Royal College of Paediatrics and Child Health annual conference |  |  |  |  |  |  |  |  |  |  |  |  |
| Cochrane Colloquium |  |  |  |  |  |  |  |  |  |  |  |  |
