# Supplementary material for: Parent-to-parent support interventions for parents of babies cared for in a neonatal unit—protocol of a systematic review of qualitative and quantitative evidence
Source: Syst Rev. 2018 Oct 31;7:179. doi: 10.1186/s13643-018-0850-2 (PMC6211448; doi:10.1186/s13643-018-0850-2)
Supplement: Supplementary file 3 — Plain Language Protocol Summary. (DOCX 6162 kb) [file 13643_2018_850_MOESM3_ESM.docx]

# Parent-to-Parent Support for Parents of Babies Cared for in a Neonatal Unit (PaReNt)


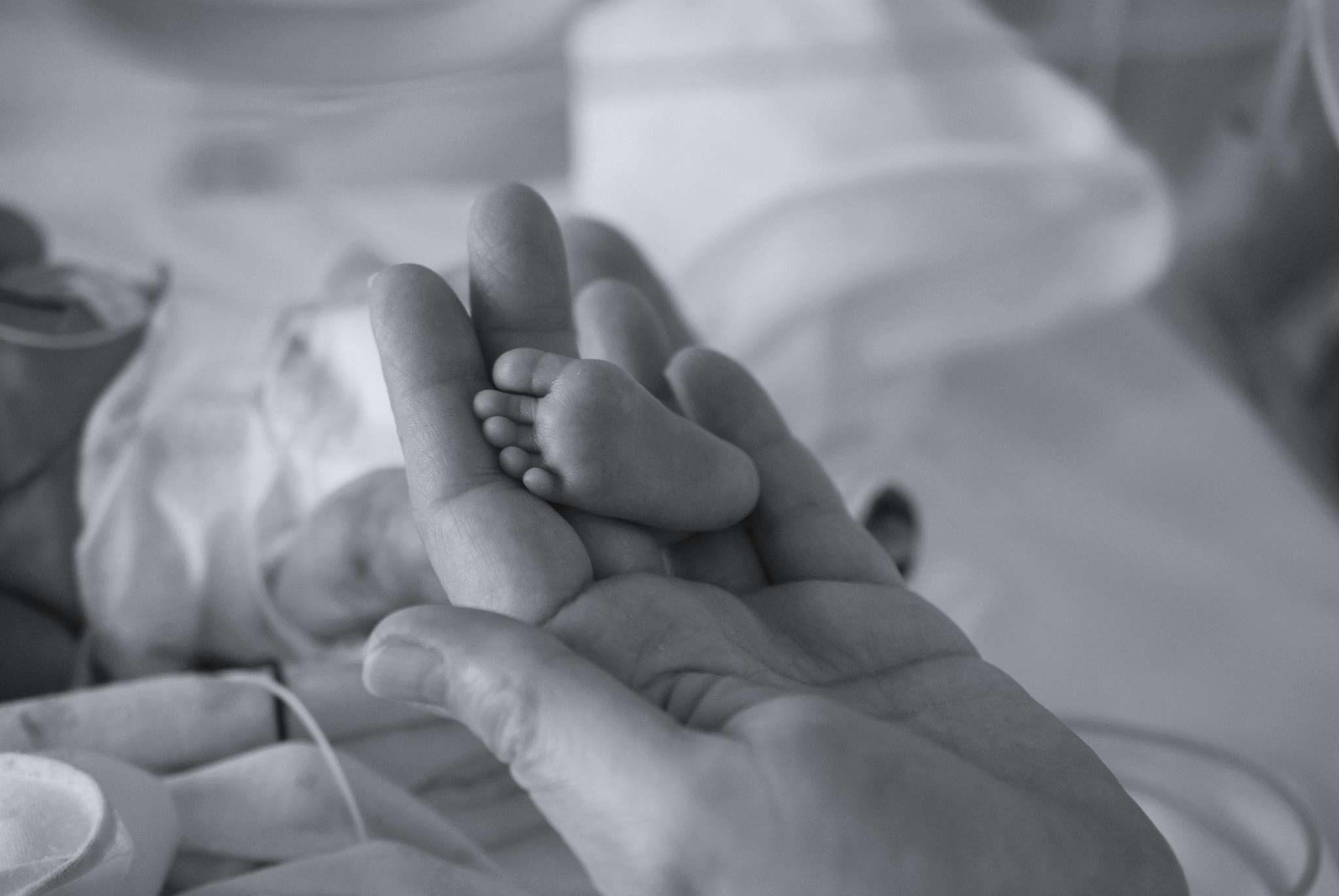


| Research team: | | 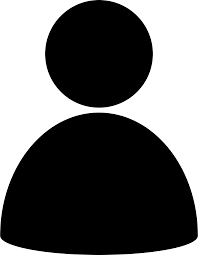 | Leanna Wakely | 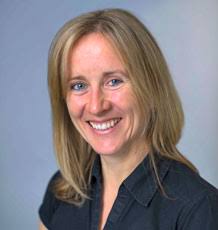 | Becca Abbott | 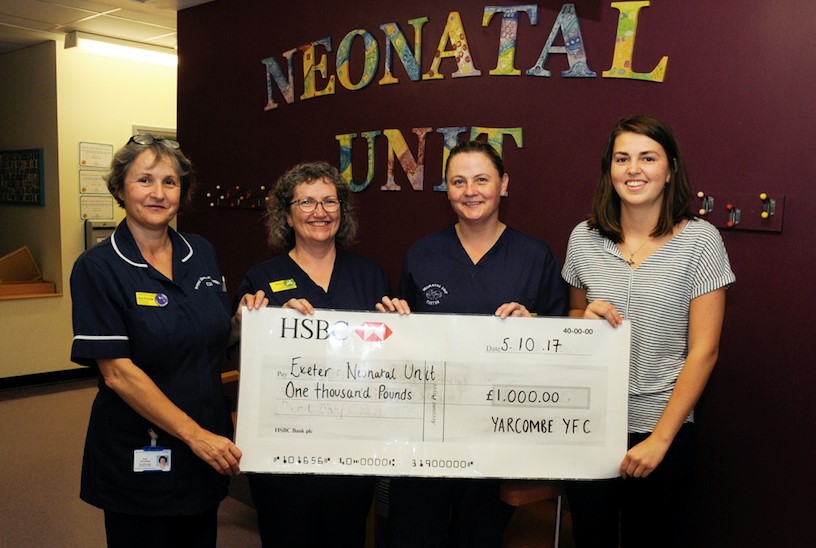 | Sue Prosser |
| --- | --- | --- | --- | --- | --- | --- | --- |
| 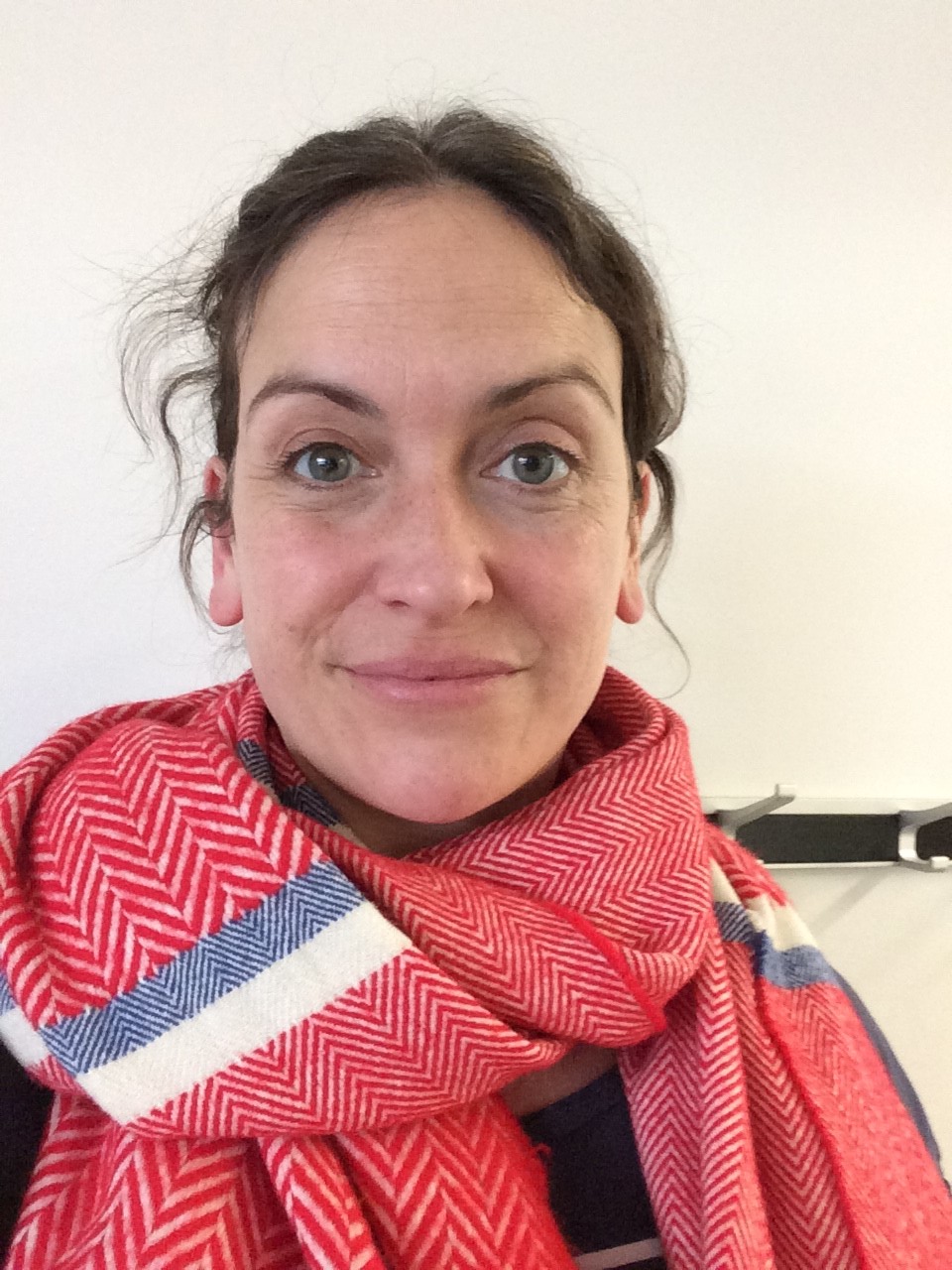 | Harriet Hunt | 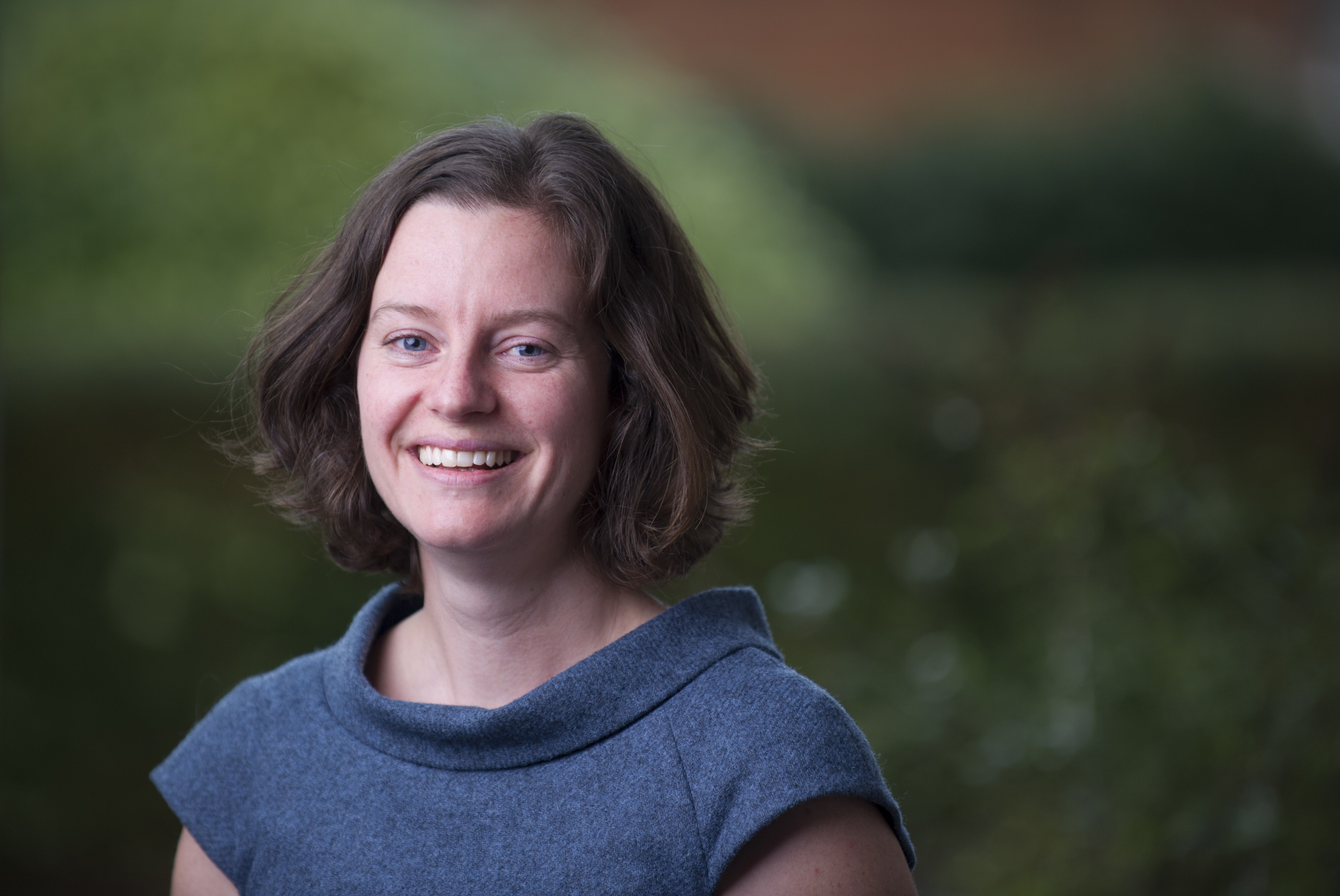 | Kate Boddy | 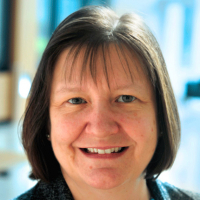 | Jenny Kurinczuk | 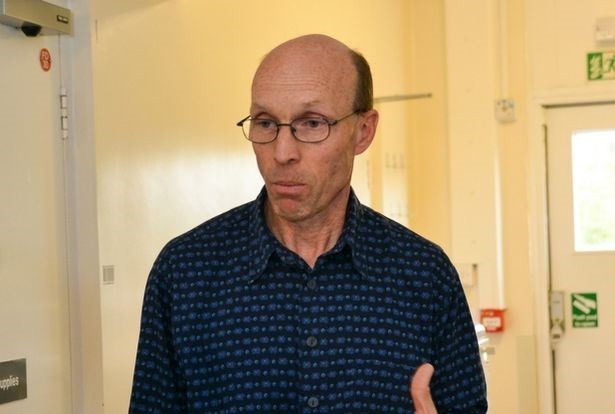 | Andrew Collinson |
| 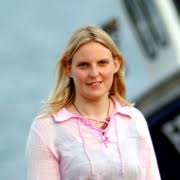 | Rebecca Whear | 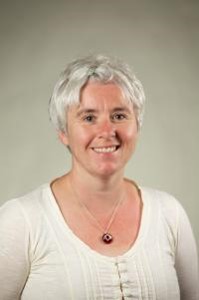 | Alison Bethel | 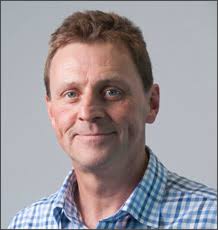 | Chris Morris | 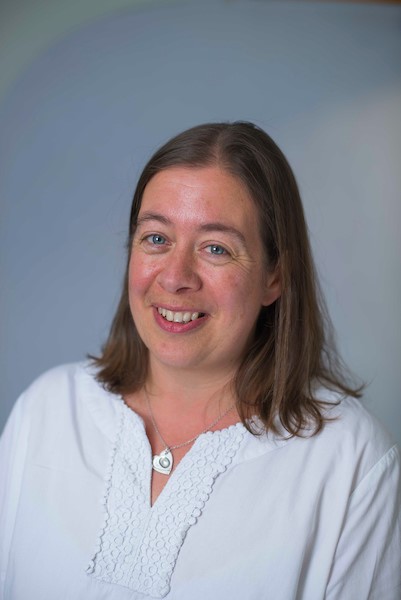 | Jo Thompson-Coon |

A protocol is an essential part of a research project. It describes in detail how the research will be carried out. This is important so that people can check whether what was done in a study was what the researchers said they would do, making the research more trustworthy.

This “plain language protocol summary” aims to explain the steps that we will go through to carry out this research. It will help anyone interested in our research to understand the project without all the details necessary in a full protocol.

## Where will this information be available?

This plain language protocol summary (PLPS) can be found, along with the published academic protocol, [on our research group website](http://clahrc-peninsula.nihr.ac.uk/research/parent-to-parent-support), on the National Institute for Health Research (NIHR) website , on the International Prospective Register for Systematic Reviews (PROSPERO CRD42018090569), in our peer-reviewed open access protocol, and as a working document for the parents involved in the project.

Parents of babies admitted to neonatal units experience an emotional journey. Feelings of helplessness, fear, sadness, guilt, grief and anger are common. These feelings can lead to anxiety, depression and post-traumatic stress which may persist long after discharge from the unit. Support from a parent with first-hand experience able to empathise with problems and challenges may help.

We want to find out:

- Whether parent-to-parent support is helpful for families experiencing neonatal care;
- what this support looks like; and
- what things can help or prevent parent-to-parent support being available.

## Who is carrying out the research?

The core team are from the University of Exeter Medical School and we have help from specialists in supporting parents of babies in neonatal care. A key member of our research team is from our charity partner SNUG (Supporting Neonatal Users and Graduates) with wide experience in parent to parent support within neonatal care. Two members of our team work in neonatal units in Exeter and Truro.

We are being advised by a group of parents with experience of neonatal care called the Parent Advisory Group (PAG). The PAG meets with the research team every 3 months to help make sure the research is relevant to parent’s lives, and our findings are likely to be more useful and accessible.

## How will we carry out the research?

The research we are doing is called a ‘systematic review’. This brings together all the research evidence that has been done on this topic already to answer our questions. The stages of a systematic review are described below and illustrated in Figure 1.


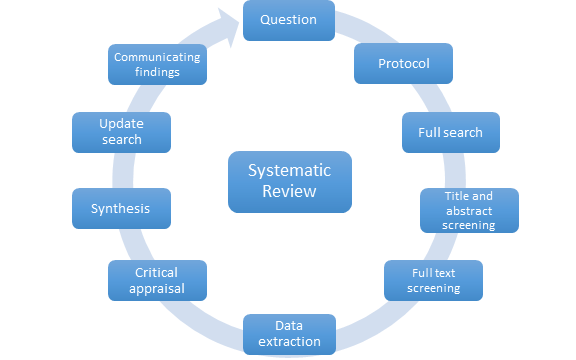


Figure 1. Stages of a systematic review

The protocol is the plan for how the systematic review will be carried out, and is produced before the research begins. This plain language protocol summary will be part of the research protocol.

Full search - Our information specialist identified the particular words that describe parent to parent support and babies in neonatal care. This was done by looking through existing studies we already knew about, and asking for ideas from our team and the PAG. These words will be used to search in databases of published evidence to identify studies.

Title and abstract screening - the full search gives a list of study titles and summaries potentially relevant to the review. The research team, and members of the PAG who would like to be involved, will read titles and summaries to see which studies might be eligible for our research. Two people will read every title and summary independently to decide if it’s relevant to our project or not, and compare answers. If they are unsure, they will include the study.

Full text screening - we will obtain and read the whole paper for each of the included studies identified, following the same process as for title and sumaary screening – two people make a decision about whether a study should be included or not in the review, then compare answers and discuss any disagreements. A third researcher will help make a final decision if it’s still not clear.

Data extraction involves gathering the same information from included studies. We will design a form to do this in a systematic way. This makes it easier to compare studies, see similarities and differences, and makes it more straightforward to find key information.

Critical appraisal is a process of judging how useful each included study is to the research question. Each paper will be examined by assessing how the research was done, what methods were used and whether it answers the question we’re asking. We will use recognised checklists judged to be sound by other systematic reviewers. Judging the quality of each study will help us prioritise findings and evidence from the more robust studies.

Synthesis is a way of combining the findings from all the studies. We expect to identify studies in which researchers have interviewed parents that have given/received support about their experiences (qualitative research) and studies in which the effects of the support on health outcomes for babies and their parents have been measured (quantitative research). Findings will be described, compared, and – where it makes sense to do so – combined.

Through this synthesis we aim to

- show the evidence we have discovered about parent-to-parent support for parents of babies cared for in neonatal units;
- look for similarities and differences in the results from included studies; and
- draw conclusions that link the success of different parent-to-parent support programs with the experience of parents.

The Parent Advisory Group will play a central role in this stage of the research, drawing on their experiences of neonatal care and peer to peer support. We will hold two PAG workshops to discuss The developing qualitative and quantitative synthesis.

Communicating findings - In the later stages of the project we will hold a conference to share our emerging findings and discuss the best ways to communicate these results. We will invite members of our PAG, staff from neonatal units, neonatal charities and other stakeholders.

Once the research is completed we will write up the study and produce conclusions and recommendations. We will use different approaches to reach different audiences for our research.

We will publish our findings in an open access, peer-reviewed journal and on social media working with our PAG. We will also present our findings at two academic conferences: the British Association of Perinatal Medicine conference taking place on 27^th^ and 28^th^ September in Leeds, and the Cochrane Colloquium on 16 to 18^th^ September in Edinburgh. We have budgeted for a member of the PAG to attend the Cochrane Colloquium and present our findings alongside a member of the core team.

## How to get in touch and find out more

Project website: <http://clahrc-peninsula.nihr.ac.uk/research/parent-to-parent-support>

🖂 [h.a.hunt@exeter.ac.uk](mailto:h.a.hunt@exeter.ac.uk)


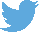
[@EvidSynthTeam](https://twitter.com/EvidSynthTeam)

##
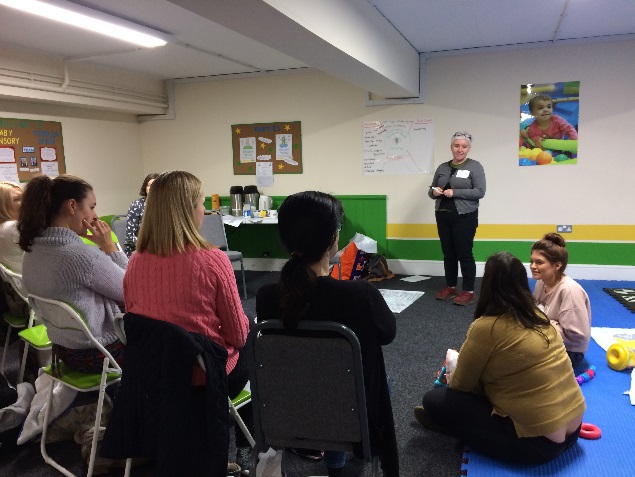


At one of our Parent Advisory Group meetings
