# Supplementary material for: Parent-to-parent support interventions for parents of babies cared for in a neonatal unit—protocol of a systematic review of qualitative and quantitative evidence
Source: Syst Rev. 2018 Oct 31;7:179. doi: 10.1186/s13643-018-0850-2 (PMC6211448; doi:10.1186/s13643-018-0850-2)
Supplement: Supplementary file 4 — Example Search Strategy. (DOCX 16 kb) [file 13643_2018_850_MOESM4_ESM.docx]

## Example search strategy

Database: Ovid MEDLINE(R) Epub Ahead of Print, In-Process & Other Non-Indexed Citations, Ovid MEDLINE(R) Daily, Ovid MEDLINE and Versions(R) <1946 to February 28, 2018>

Search Strategy:

--------------------------------------------------------------------------------

1 peer.tw.

2 parent*.tw.

3 befriend*.tw.

4 face to face.tw.

5 one to one.tw.

6 mum$1.tw.

7 mother*.tw.

8 dad$1.tw.

9 father*.tw.

10 caregiver*.tw.

11 group*.tw.

12 network*.tw.

13 program*.tw.

14 meeting*.tw.

15 match.tw.

16 friendship.tw.

17 neonat*.tw.

18 nicu.tw.

19 scbu.tw.

20 Special care baby unit*.tw.

21 nnu.tw.

22 adjusted age.tw.

23 corrected age.tw.

24 exp Intensive Care, Neonatal/

25 exp Intensive Care Units, Neonatal/

26 premature.tw.

27 preterm.tw.

28 pre term.tw.

29 weeker.tw.

30 low birth weight.tw.

31 lbw.tw.

32 vlbw.tw.

33 baby.tw.

34 babies.tw.

35 infan*.tw.

36 newborn*.tw.

37 graduate*.tw.

38 survivor*.tw.

39 *Self-Help Groups/

40 1 or 2 or 3 or 4 or 5 or 6 or 7 or 8 or 9 or 10

41 11 or 12 or 13 or 14 or 15 or 16

42 ((peer or parent* or befriend* or face to face or one to one or mum$1 or mother* or dad$1 or father* or caregiver*) adj2 (group* or network* or program* or meeting* or match or friendship)).tw.

43 support*.tw.

44 mentor*.tw.

45 (support* adj2 (group* or network* or program* or meeting* or match or friendship)).tw.

46 41 or 43

47 (mentor* adj2 (group* or network* or program* or meeting* or match or friendship or support*)).tw.

48 ((peer or parent* or befriend* or face to face or one to one or mum$1 or mother* or dad$1 or father* or caregiver*) adj2 support*).tw.

49 ((peer or parent* or befriend* or face to face or one to one or mum$1 or mother* or dad$1 or father* or caregiver*) adj2 mentor*).tw.

50 39 or 42 or 45 or 47 or 48 or 49

51 26 or 27 or 28 or 29 or 30 or 31 or 32

52 33 or 34 or 35 or 36 or 37 or 38

53 ((premature or preterm or pre term or weeker or low birth weight or lbw or vlbw) adj2 (baby or babies or infan* or newborn* or graduate* or survivor*)).tw.

54 17 or 18 or 19 or 20 or 21 or 22 or 23 or 24 or 25 or 53

55 50 and 54
