# Supplementary material for: Parent-to-parent support interventions for parents of babies cared for in a neonatal unit—protocol of a systematic review of qualitative and quantitative evidence
Source: Syst Rev. 2018 Oct 31;7:179. doi: 10.1186/s13643-018-0850-2 (PMC6211448; doi:10.1186/s13643-018-0850-2)
Supplement: Supplementary file 5 — Plain language summary. (PDF 315 kb) [file 13643_2018_850_MOESM5_ESM.pdf]

PaReNt

WE'RE CARRYING OUT A RESEARCH PROJECT ABOUT PARENT-PARENT SUPPORT IN NEONATAL CARE – WOULD YOU LIKE TO HELP?

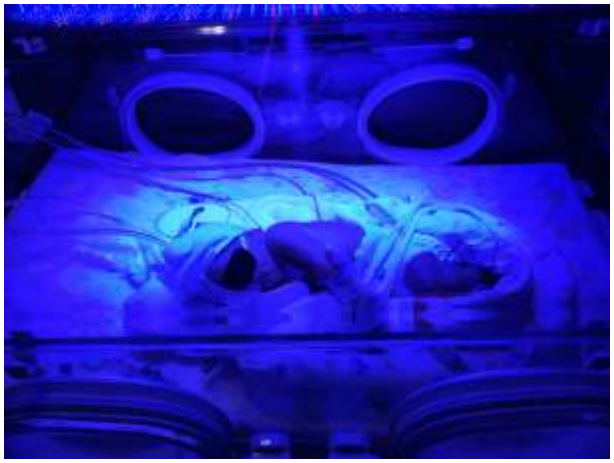

WHAT IS PaReNt?

Parents of babies admitted to neonatal units experience an emotional journey. Feelings of helplessness, fear, sadness, guilt, grief and anger are common and may persist long after leaving the hospital. Support from a parent peer who has been through the same experience and can empathise with the challenges may help. Researchers from the University of Exeter Medical School and Royal Devon and Exeter Foundation Trust, the Royal Cornwall Hospitals NHS Trust and local charity SNUG (Supporting Neonatal Users and Graduates), want to find out if peer support is effective for helping families during neonatal care. We will also examine what peer support looks like, how it is organised and what the impacts and challenges are to implementing this kind of support.

WHAT WILL BE HAPPENING?

The research we will be doing is called a 'systematic review'. This brings together all existing research on a particular topic. To find studies that might help us to answer our research question, we search for relevant published research studies. We will also ask clinicians, researchers and parents and look at websites of relevant organisations to identify any other useful information. We will look for similarities and differences in the results we find from research studies we identify and will draw conclusions about the effectiveness of peer support with parents' experience.

HOW WILL THIS HELP?

During the project we will bring together local and national neonatal service representatives, such as health staff and charities, to discuss the findings of the review and how they can be used to ensure this work has an impact on health care services and family outcomes and guide any future research in this area.

Royal Devon and Exeter NHS
NHS Foundation Trust

SNUG

UNIVERSITY OF EXETER | MEDICAL SCHOOL

Royal Cornwall Hospitals NHS
NHS Trust

WHAT WOULD YOUR ROLE BE IN OUR PUBLIC INVOLVEMENT GROUP?

We are seeking to work with a small group of parents who have had experience of neonatal care. You would be an advisor to the research team. Your advice will help ensure the research is relevant to parent's lives, and the findings are likely to be more useful and accessible. You do not need to have knowledge about research and your participation can be flexible to suit changing commitments.

Public and patient involvement is a partnership between patients and the public and researchers in the research process, it is defined as doing research 'with' people who use services rather than 'to', 'about' or 'for' them.

HOW WILL THE GROUP CONTRIBUTE TO THE PROJECT?

- By giving feedback on our work.
- Keeping the research relevant to parent's lives by sharing your experiences.
- Participating in group meetings, which will be about 2 hours with light refreshments provided.
- By reading and commenting on documents by e-mail or telephone occasionally.

WHAT CAN YOU EXPECT FROM RESEARCHERS?

- To be welcomed, valued and listened to respectfully.
- To be able to contact a member of staff before and after each meeting.
- To have paper copies of any research documents sent out in advance of meetings.
- To have meetings arranged within school hours and at a convenient and child-friendly location.
- To have any accessibility or additional needs taken into account.
- To be kept up to date with research progress at least every two months and to receive feedback on meetings attended.

WHAT ABOUT YOUR CHILDREN?

We plan to hold the meetings at a very child friendly venue and run them in such a way that you can bring your children with you and they will be very welcome. We can also cover some childcare costs if you prefer to come without them, please just discuss this in advance.

IS THERE ANY PAYMENT?

You will receive a 'thank you' payment to acknowledge your time (usually £25, depending on the length of meeting). We will also reimburse any travel and childcare expenses (must be Ofsted Registered) that you have in order to attend the meetings.

KEEPING IN TOUCH

Between meetings we will communicate with the group by email, or letter if preferred, to provide project updates and consult about specific issues as appropriate. We also intend to use social media such as Facebook or WhatsApp to make communication easier but this will be based on what the group prefers.

Kate Boddy will be the first point of contact about the project for queries etc from group members.

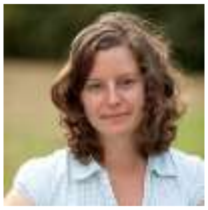

K.boddy@exeter.ac.uk
01392 726065

Funded and supported by NHS National Institute for Health Research
