# Supplementary material for: Parent-to-parent support interventions for parents of babies cared for in a neonatal unit—protocol of a systematic review of qualitative and quantitative evidence
Source: Syst Rev. 2018 Oct 31;7:179. doi: 10.1186/s13643-018-0850-2 (PMC6211448; doi:10.1186/s13643-018-0850-2)
Supplement: Supplementary file 6 — Fact finding phonecalls topic guide. (DOCX 21 kb) [file 13643_2018_850_MOESM6_ESM.docx]

## Fact finding phone calls topic guide

Who will be conducting these phone calls?

Sue Prosser will make the phone calls using her professional network of staff from the 12 neonatal units within the South West.

Harriet Hunt will assist by note taking and clarifying any project details during or after the phone calls. We have allocated two timeslots to make these calls on Thursday 22^nd^ February 1-4pm and Monday 26^th^ February from 11.00 to 1.30pm in Sue’s office at the RD&E in Exeter.

What’s the aim of these phone calls?

We will talk to relevant contacts in each of the 12 South West Neonatal units to establish

- what (if any) parent-to-parent support services are provided on their unit,
- what those services look like,
- who provides them,
- what barriers or facilitators have been seen in implementation and
- any concerns about service sustainability.

How will this information be used?

We will use this work alongside any other similar work conducted in the UK to more fully inform the discussion during the impact conference and the subsequent key messages to be promoted from the research findings.

Fact finding calls – how will they go?

*Quick introduction to the project*

This is a project looking at parent-to-parent (sometimes called ‘peer’) support provided to parents of babies cared for in neonatal units. We’re very specifically focussed on parents supporting other parents with babies cared for in neonatal units, and this can be in person, over the phone, online – any way.

The main focus of the project is to explore what services are available, and what works and doesn’t work. We will be doing this via a systematic review looking at all the existing evidence on this topic. During the project we will bring together local and national neonatal service representatives, such as health staff and charities, to discuss the findings of the review and how they can be used to ensure this work has an impact on health care services and family outcomes and guide any future research in this area. We would like you to be involved in this and it would make a real difference to our work.

*Questions*

1. What parent-to-parent support services are provided on your unit? (give examples if needed)
2. What do those services look like? (e.g. Are they offered all the time or just to some people? How do you decide who needs what? Is there one person in charge of a particular service?)
3. Who provides these services? (e.g. How are they staffed? Are they provided in-house, contracted out or run by volunteers? How are they funded?)
4. What helps the services work? What doesn’t work? Are there any examples of services that have closed? Are there any services that have done well?
5. Do you have any worries about the services running into the future? How will they keep going?
6. Do you have any other comments or questions about parent-to-parent support services that you provide or have come across?

Concluding the fact finding phone calls

We will be in touch with our contacts from each of the 12 units to let them know how the project is progressing, and to invite people along to our South West impact conference in July/September 2018. The aim of this is to discuss what we have found, what the really important messages are and how to get these out to where they are most needed.

If we can finish the call with answers to the questions above plus the contact details of one or two people from each unit who would like to see our results and help shape our key messages we’d be really grateful.

## Questions with additional items suggested by PAG in italics

| **What parent-to-parent support services are provided on your unit?**  *Do you run neonatal coffee mornings?  Prompt: do you have parent volunteers visiting parents at the cotside? Are the parents registered hospital volunteers?*  ***How are parents informed about parent to parent support?*** ***When babies are discharged from the NNU are the parents reminded about the parent to parent support?*** |
| --- |
| **What do those services look like?** (e.g. Are they offered all the time or just to some people? How do you decide who needs what? Is there one person in charge of a particular service?)  *e.g. neonatal coffee mornings:*  *who leads on these? Is the designated person running these a parent or a staff member?*  *How regularly do they occur?*  *Are they well attended/ Do you record how many attend?*  *Are they accessible to parents? What time do they take place? Is during ward rounds or visiting times?  Are ex neonatal users welcome?*  ***Does your unit have a peer support Facebook group?*** *Who runs this? Are staff involved in this? What is the impact of online support for parents?* |
| **Who provides these services?** (e.g. How are they staffed? Are they provided in-house, contracted out or run by volunteers? How are they funded?)  *parent volunteer concept:*  *How many parents come to you per year and ask about volunteering to support parents?   Is there a process in place for managing this?  Is there a member/members of staff assigned to manage volunteers?  How are volunteers supported?  How frequently is parent volunteer support reviewed?*  ***Does your unit link to any neonatal peer support groups in the community?*** *Who leads on this? How closely does the unit work with the community support group?* |
| **What helps the services work? What doesn’t work? Are there any examples of services that have closed? Are there any services that have done well?**  *What are the barriers to implementing peer supporters on the unit?   What do you think the issues and challenges are around implementing peer support? What are the barriers to running coffee mornings?* |
| **Do you have any worries about the services running into the future? How will they keep going?** *Are you happy with the current level of peer supporters working with the unit?* |
| **Do you have any other comments or questions about parent-to-parent support services that you provide or have come across?** |
